# Solidification Morphology and Bifurcation Predictions with the Maximum Entropy Production Rate Model

**DOI:** 10.3390/e22010040

**Published:** 2019-12-26

**Authors:** Yaw Delali Bensah, J. A. Sekhar

**Affiliations:** 1 Department of Materials Science and Engineering, University of Ghana, Legon, Accra P.O. Box LG 77, Ghana; bensahyad@gmail.com; 2 Department of Mechanical and Materials Engineering, University of Cincinnati, Cincinnati, OH 45221, USA

**Keywords:** maximum entropy production rate, MEPR, planar morphology, cellular morphology, morphological bifurcations at solid–liquid interface, growth velocity, temperature gradients, coefficient of diffusion at high temperatures

## Abstract

The use of the principle of maximum entropy generation per unit volume is a new approach in materials science that has implications for understanding the morphological evolution during solid–liquid interface growth, including bifurcations with or without diffuseness. A review based on a pre-publication arXiv preprint is first presented. A detailed comparison with experimental observations indicates that the Maximum Entropy Production Rate-density model (MEPR) can correctly predict bifurcations for dilute alloys during solidification. The model predicts a critical diffuseness of the interface at which a plane-front or any other form of diffuse interface will become unstable. A further confidence test for the model is offered in this article by comparing the predicted liquid diffusion coefficients to those obtained experimentally. A comparison of the experimentally determined solute diffusion constant in dilute binary Pb–Sn alloys with those predicted by the various solidification instability models (1953–2011) is additionally discussed. A good predictability is noted for the MEPR model when the interface diffuseness is small. In comparison, the more traditional interface break-down models have low predictiveness.

## 1. Introduction

The maximum entropy generation principle [1,2,3,4,5,6,7,8,9,10,11,12,13,14,15,16] has brought significant predictive capability to quantitative materials science. The principle has been able to reveal (a) the onset of various forms of morphological bifurcations during growth, especially the onset of the first roughening transition [1,2,6,9]; (b) complex solute segregation and texture reconfiguration phenomena, particularly noted during tribological contacts [4,12]; (c) Belousov–Zhabotinsky patterns and reaction pathways for high temperature reactions [2,3]; (d) the onset of steady state structures during solid–solid wear [5,6,7,8]; and (e) the stable ellipsoidal patterns that are noted in solidification microstructures (dendrite tips), multiphase fluid flows, and particle sedimentation [2,10,11,12,13,14,15,16,17,18,19]. The principle accurately predicts the trajectory of objects in motion when subjected to a gravitational field [16] and by extrapolation of the field-theory for the assessment of the efficacy of solar cells [17].

The maximum entropy production rate-density, MEPR, or the Maximum Entropy Production Principle, MEPP, are the acronyms used in the literature [2,6,9,10] for analyses that employ the entropy rate maximization principle. We have chosen to use MEPR [2] in this article to emphasize the importance of the “rate” in the acronym. A MEPR hypothesis is tested in this article for initiating interface diffuseness or topographical changes in the two-phase and/or diffuse interface regions. Any criterion for bifurcation based on this principle is expected to be related to the composition, partition function, velocity of solidification, and the temperature gradient experienced in the solid–liquid zone [1,2,18].

The MEPR model postulates that the entropy generation is maximized for an interface transition (bifurcation) to a different configuration (i.e., to a different atomistic or topographical (morphological) variant). In this article, we first review the MEPR solidification bifurcation model [1,2] and compare it with other models for the plane front instability. The model predictions are tested with the experimental data available from published studies on numerous dilute binary alloys [1]. The ensuing model for pure substance or a binary alloy has been shown to quantitatively predict the size of a diffuse interface and the number of pseudo-atomic layers present in the diffuse zone [1]. This is reviewed below. A further comparison with experimentally measured breakdown groupings of variables that have been reported in the solidification-research literature indicates that the MEPR model is also able to account for the interface topography as being either of a faceted (f) or non-faceted (n/f) kind [1]. It must be noted that model also correctly offers a quantitative measure for the transition from facet to non-facet (f/nf) planar or to a non-planar topography, *as being dependent on the velocity and the temperature gradient* [1]. As the entropy generation is related to the gradients, it is possible that the MEPR criterion may also allow for a better estimate of the solute diffusion constant in binary alloys [18] that has proved elusive to predict by traditional models. This hypothesis is tested for dilute Pb–Sn alloys in this article.

When an alloy is directionally solidified at a low velocity (typically in the order of one micrometer per second), a planar morphology is first noted at the solid–liquid interface. With an increase in the transformation velocity (enabled by increasing the cooling rate or the Bridgman–Stockbarger imposed growth rate (See for example https://en.wikipedia.org/wiki/Bridgman-Stockbarger_technique), the initially planar interface becomes unstable with other shapes, transforming to a non-planar, macroscopically jagged or wavy cellular shape to the variations possible in the topography; or it may transform to a diffuse interface with non-planar shape variations [18]. There is a loss of work potential, W_L_, with a new shape or diffuseness formation, which in turn is related to the entropy generation. The appearance of a smooth-cellular or jagged morphology from a planar interface, especially for binary alloys, depends on the material composition, C_O_ (wt% or mole/m^3^), velocity V (m/s) of the growing interface, and the temperature gradient G_L_ (K/m) in the liquid and k, the non-dimensional solute partition coefficient. These variables at the point of morphological instability are commonly subscripted with the symbol (c) to indicate a transition [18,19,20,21,22,23,24,25,26,27,28,29,30,31,32,33,34,35,36,37,38,39,40,41,42]. Various theoretical models [18,19,20,21,22,23,24,25,26,27,28,29,30,31,32,33,34] have been offered to explain the transition, however, the two most widely employed models (prior to MEPR) that describe the interface instability from planar to non-planar are the constitutional undercooling (CUT) [19,20,21,22,27] and the linear stability theory model (LST) [31]. A minimum–maximum entropy model was also previously proposed [24] but did not provide comprehensive predictability, ostensibly because the Prigogine criterion used in that model for the minimum–maximum criterion was not applicable [2]. The objective of this article is to extend the confidence in the MEPR model with comparative tests made in [1] in order to select between the models.

The CUT, LST, and MEPR models contain a diffusion parameter, namely, the coefficient of diffusion of solute in the liquid. Consequently, one additional test for the comparison of the three models is to compare the predicted values of the coefficient of diffusion to an experimental number measured from a *non-solidification* experiments at that temperature. In [18], it was shown that the values predicted by CUT and LST models show considerable deviation from the experimental number. In this article, comparisons with the MEPR model are made.

## 2. The MEPR Model

During the one dimensional solidification of a pure metal or a binary molten alloy, which is at freezing temperature under a fixed temperature gradient and with constant interface velocity there is a loss of work potential from the dissipation of kinetic energy, giving rise to entropy generation rate density ${\dot{\varphi }}_{\max}$ (J m^−3^K^−1^s^−1^) is given by [1] in the region of the diffuse interface with dimensions ζ (m). The subscript max. indicates that that the maximum value of this new entropy generation rate is given by:

(1) $${\dot{\varphi }}_{\max}=\frac{\Delta \rho_{k}{\;V}^{3}}{2{\;\zeta }^{2}{\;G}_{\mathrm{SLI}}}$$

where Δ*ρ*_k_ (kgm^−3^) is the overall density shrinkage given by (*ρ*_l_ Δ*ρ*/*ρ*_s_), Δ*ρ* (kgm^−3^) is the density change from liquid to solid (*ρ*_s_ − *ρ*_l_); *ρ*_s_ (kgm^−3^) and *ρ*_l_ (kgm^−3^) are the densities of the fully solid and fully liquid zones, respectively. The symbol G_SLI_ (Km^−1^) is the temperature gradient across the solid–liquid interface, including the diffuse interface. This gradient is difficult to measure experimentally, so it is commonly approximated as the average between the rigorous-solid and rigorous-liquid regions. In this article, G_SLI_ is assumed to be approximately equal to the temperature gradient in the fully liquid zone, G_L_ (Km^−1^), (i.e., G_SLI_ ≈ G_L_).

At the solid–liquid interface region, during directional solidification of a binary material, the existence of diffuseness or a non-planar morphology (such as cellular) can produce new entropy. Following Sekhar [2], the entropy rate maximization in this region when compared between various morphological pathways can be thought to be somewhat analogous to the free energy selections between various phases. A cellular structure produces entropy of a positive value that increases with velocity, as does the planar diffuse structure [1,2], but at different rates. The postulate of MEPR applied to solidification morphology is that the highest entropy-rate-producing configuration is the most stable. During directional solidification (one dimensional growth in casting, as is done for turbine blades or jewelry manufacture), the first transition from a stationary planar interface is the evolution in the interface region from an atomically sharp to a diffuse interface between the rigorous solid and the rigorous liquid [2]. For an alloy, further topographical variations become possible as the entropy generation rate per unit volume reaches a peak, beyond which a cellular or other non-planar structure (e.g., cells or dendrites) can overtake the planar entropy production rate at any given composition of the alloy [1,2]. Detailed calculations for developing Equation (1) for the diffuseness dimension and the instability criterion are shown in [1]—only a brief review is provided below. The maximum entropy generation rate per unit volume (or the entropy rate density) [1,2] is related to ${\dot{s}}_{\mathrm{LG}}($Jm^−3^K^−1^s^−1^), which is the entropy transfer rate from the solute gradient in the liquid and ${\dot{s}}_{E}$(Jm^−3^K^−1^s^−1^) (i.e., the main component of the entropy generation rate that describes the entropy generated due to exchange of matter and heat in the SLI), expressed as:

(2) $${\dot{\varphi }}_{\max}\;={\dot{s}}_{E}-{\dot{s}}_{\mathrm{LG}}$$

${\dot{s}}_{E}$ and ${\dot{s}}_{\mathrm{LG}}$ are given by $\left(V\Delta h_{\mathrm{sl}}{\;G}_{\mathrm{SLI}}/T_{M}^{2}\right)$ and $\left(\Delta T_{O}V^{2}R_{g}\ln\left(1/k\right)/D_{L}\;4{\;m}_{L}\right)$, respectively [1]. The term ∆h_sl_ (Jm^−3^) is the heat of fusion, which is an approximation for ∆h_m_ [2]; ∆h_m_ (Jmol^−1^) is the heat of fusion with defects; m_L_ (Km^3^mole^−1^) is the slope of the liquidus line at the solid–liquid boundary for a binary material; k (dimensionless) is the partition coefficient that can be obtained from the binary phase diagram; D_L_ (m^2^s^−1^) coefficient of solute diffusion in the alloy. Here, ΔT_O_ (K) is the solidification temperature range, which is expressed as (T_l_ − T_s_) or (C_O_(1 − k) m_L_/k), where T_l_ and T_s_ are the liquidus and solidus temperatures, respectively, and can be obtained from the phase diagram. The conditions given by Equations (3a) and (3b) below for a maximum or minimum defines a possible onset of a bifurcation condition (morphological instability).

(3a) $${\left(\frac{\partial {\dot{\varphi }}_{\max}}{\partial V}\right)}_{\;C_{O}}=0$$

or

(3b) $${\left(\frac{\partial {\dot{\varphi }}_{\max}}{\partial V}\right)}_{\zeta \;}=0$$

Note that arguments to indicate the maximization condition are provided in [2,6,9,10]. Note that ${\left(\frac{\partial^{2}{\dot{\varphi }}_{\max}}{\partial V^{2}}\right)}_{\zeta ,{\;C}_{O}}$ is negative when inferring a maximization condition. Although T_si_ and T_li_ are unknown based on binary alloy materials, the thickness ζ of the diffuse interface (*m*) can be approximated for dilute solutions by assuming that T_si_ ≈ T_m_ and T_li_ ≈ T_m_, and following the procedures developed in [1], the standard solute balance at steady state growth along with Equation (3a) above can used to yield:

(4) $${\left(\frac{\partial {\dot{\varphi }}_{\max}}{\partial V}\right)}_{C_{O}}=\;\frac{\;\Delta h_{\mathrm{sl}}{\;G}_{\mathrm{SLI}}\;}{T_{\mathrm{li}}\;\cdot {\;T}_{\mathrm{si}}}-\frac{2\Delta T_{O}}{D_{L}}\frac{{\;V\;R}_{g}\ln\left(1/k\right)}{4{\;m}_{L}}$$

Here, C_O_ (wt % or mole m^−3^) is the solute concentration in the alloy.

Similarly, Equation (2) yields,

(5) $${\left(\frac{V\;}{G_{\mathrm{SLI}}\;}\right)}_{C}=\frac{D_{L}}{\Delta T_{O}}\frac{2{\;m}_{L}\;\Delta h_{\mathrm{sl}}}{T_{m}^{2}{\;R}_{g}\;\ln\left(1/k_{\mathrm{eff}}\right)}$$

Now by using (3b) and (5), ζ can be written as:

(6) $$\zeta =\frac{V}{G_{\mathrm{SLI}}}\;\frac{1}{\sqrt{M-B}}$$

where **N** (m^2^ K^−2^s^−2^) is defined as $\left[\left(\frac{\;2\Delta h_{\mathrm{sl}}}{\;\Delta \rho_{k}{\;T}_{m}^{2}}\right)-\left(\frac{V\;\Delta T_{O}{\;R}_{g}\ln\left(\frac{1}{k_{\mathrm{eff}}}\right)}{2{\;G}_{\mathrm{SLI}}{\;D}_{L}\;\Delta \rho_{k}{\;m}_{L}}\right)\right]$, **M** (m^2^ K^−2^s^−2^) is defined as $\left(\frac{\;2\Delta h_{\mathrm{sl}}}{\;\Delta \rho_{k}{\;T}_{m}^{2}}\right)$ and **B** (m^2^ K^−2^s^−2^) is defined as $\left(\frac{V\;\Delta T_{O}{\;R}_{g}\ln\left(\frac{1}{k_{\mathrm{eff}}}\right)}{2{\;G}_{\mathrm{SLI}}{\;D}_{L}\;\Delta \rho_{k}{\;m}_{L}}\right)$.

Here, k_eff_ is the effective partition coefficient for a diffuse interface. The equation is valid for extremely dilute alloys. Changing the formulation of Equation (6) by placing back into Equation (5) now also gives the *driving force diffuseness* for a binary alloy material as: $\eta_{G}=\frac{V\;}{G_{\mathrm{SLI}\;}}\;\frac{1}{d}\;\frac{1}{\sqrt{N}}$, where d is the interplanar lattice spacing normal to the growth direction. However, note that the exact bifurcation may occur at any velocity and temperature gradient greater than that set by Equation (5) (i.e., Equation (5) only sets one boundary condition). By analyzing the entropy generation density for a wavy interface [1], one can also infer that the interface will break down between

(7) $$\frac{D_{L}}{\Delta T_{O}}\frac{2{\;m}_{L}\;\Delta h_{\mathrm{sl}}}{T_{m}^{2}{\;R}_{g}\;\ln\left(1/k_{\mathrm{eff}}\right)}<{\left(\frac{V\;}{G_{\mathrm{SLI}}\;}\right)}_{C}<\frac{2D_{L}}{\Delta T_{O}}\frac{2{\;m}_{L}\;\Delta h_{\mathrm{sl}}}{T_{m}^{2}{\;R}_{g}\;\ln\left(1/k_{\mathrm{eff}}\right)}$$

This is the MEPR condition for describing the breakdown limits. Because the condition is based on the comparison of the entropy rate maximization, it may also be recast in terms of the cooling rate (VG_SLI_)_C_.

**Figure 1:**
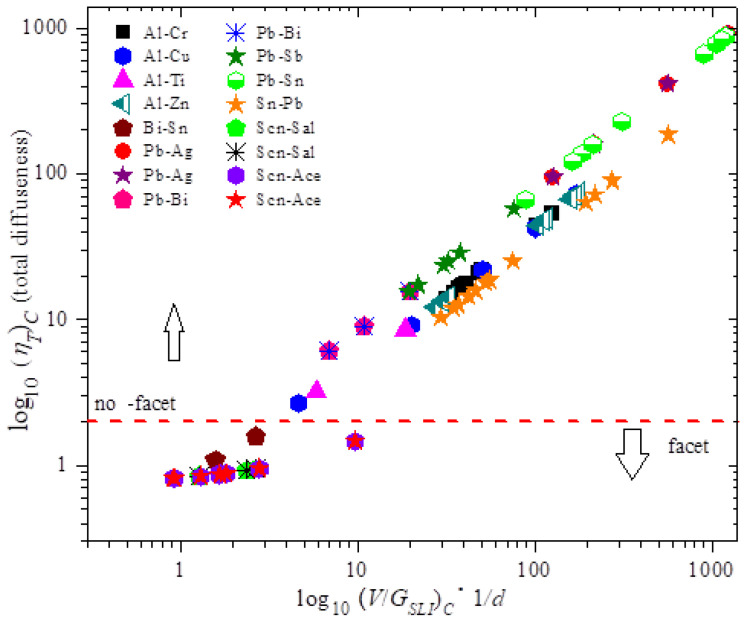
The plot shows measured experimental conditions at breakdown in the abscissa and calculated interface diffuseness on the ordinate. If the total interface diffuseness is greater than one or two atomic layers, then there is a possibility of non-facet morphology at breakdown, otherwise it should be facet morphology [1]. The relationship between total diffuseness and the ratio of the velocity/temperature gradient (V)_C_/(G_SLI_)_C_ should yield a straight line irrespective of material parameters for any growth direction (or crystal plane spacing normal to a growth direction) in the MEPR model. The values V and G_SLI_ are experimentally measured numbers at breakdown, and η_T_ is calculated from the model [1]. Note that succinonitrile (SCN) alloys are non-faceted by an additional thermal and possibly rotational diffuseness at the melting temperature, which makes the SCN material transformation always appear with a non-faceted morphology particularly when observed at optical level magnifications. Experimentally, the materials shown below the dashed line (log_10_ η_T_ = 2) are recorded to be macroscopically faceted [1]. For the zone for facet materials, a different slope than in the non-facet region may represent different mechanisms for growth (e.g., nucleation-dominated or dislocation-dominated) [22,32,43].

**Figure 2:**
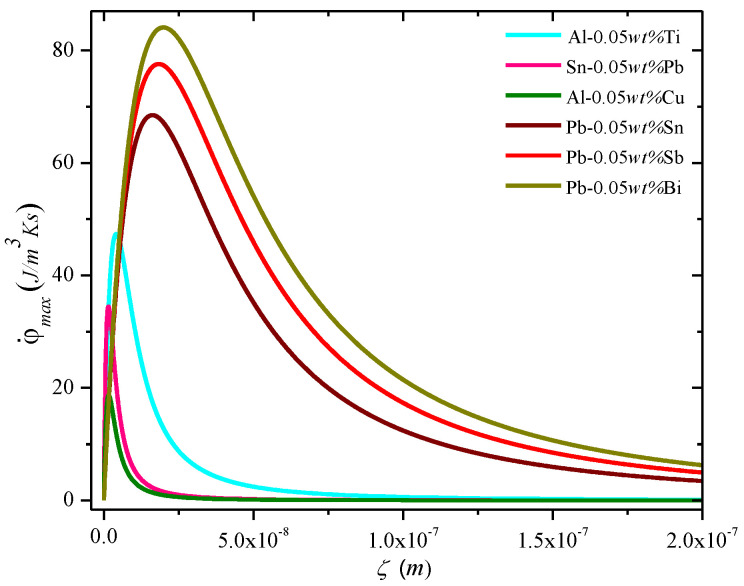
MEPR model prediction of the calculated maximum entropy generation rate density ${\dot{\varphi }}_{\max}$ (J/m^3^Ks) against the diffuse interface thickness at a constant solute concentration for dilute binary materials. The ${\dot{\varphi }}_{\max}$ for the diffuse plane front reaches its highest value at the peak of the curve (i.e., when **M** becomes equal to 2**B**).

**Figure 3:**
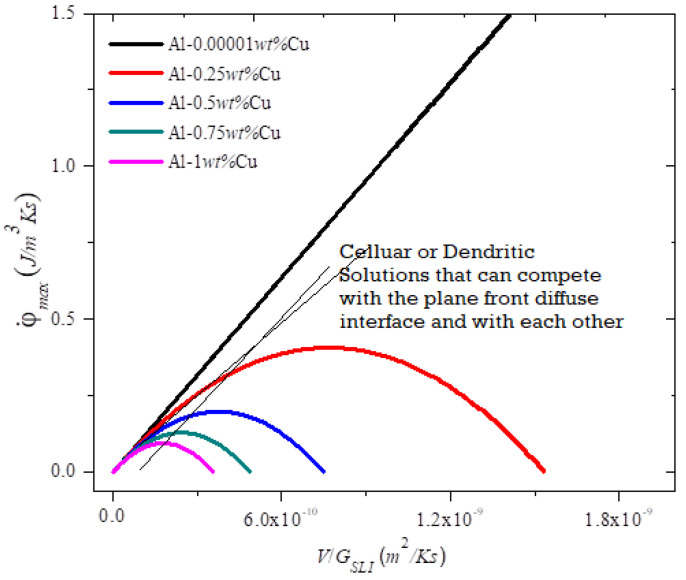
MEPR model prediction [1] of calculated ${\dot{\varphi }}_{\max}$ (J/m^3^Ks) as a function of the (V/G_SLI_) for Al–Cu with solute concentrations for five compositions (thick colored curves). The ${\dot{\varphi }}_{\max}$ increases with decreasing solute concentration and reaches a maximum value. At extremely low solute concentration, the binary material behaves similarly to a pure material (linear dark line) and ${\dot{\varphi }}_{\max}$ increases indefinitely with V/G_SLI_, like a pure metal [1]. Note the two thin schematic lines, one for cells (that begins at the origin) and the other for some form of dendrites, are also shown to indicate how a bifurcation transition may be reached, and further how dendrites can overtake cellular morphologies (see reference [2] for more details on types of dendrites). Note that in Equation (8) a similar graphical relationship for the entropy rate density generation density is seen when the abscissa is the cooling rate (V∗G_SLI_) [1]. For unconstrained dendrites [18] the cooling rate is a preferred grouping of processing variables to indicate particularly the fineness of the secondary dendrites with increased cooling rate [32,33,43,44].

Note that Figure 1 establishes a relationship between the diffuseness and the break down variable. Figure 1 shows the plot of the total diffuseness as a function of (V/G_sli_)_c_. The figure plots the set of measurable breakdown parameters. For any alloy this would be a straight line as per the MEPR model. However, we note that that the band is the similar across various alloys thus highlighting the previously unanticipated relationship between interface diffuseness and the solidification parameters. This implies that the results shown below in Figure 2 and Figure 3, namely the maximums in the entropy generation rate, are anticipated by the experiments. Additionally, the slopes are different for faceted materials when compared to the non-facet situation, possibly indicating features of diffuseness not fully captured by the MEPR model.

Equation (6) can be related to the processing parameters for constrained or unconstrained solidification namely, $\left(V/G_{\mathrm{SLI}}\right)\;$or the cooling rate (${\mathrm{VG}}_{\mathrm{SLI}})\;$respectively to yield the following entropy rate based criteria,

(8) $${\left(\frac{V}{G_{\mathrm{SLI}}}\right)}_{C}\;=\;\frac{2}{\Delta \rho_{k}\;}\;{\left(\frac{{\dot{\varphi }}_{\max}}{{N\;G}_{\mathrm{SLI}}^{2}}\right)}_{C}$$

(9) $${\left({\mathrm{VG}}_{\mathrm{SLI}}\right)}_{C}\;=\;\frac{2\;{\left({\dot{\varphi }}_{\max}\right)}_{C}\;}{\Delta \rho_{k}{\;N}_{C}}$$

where c refers to critical and N is defined below Equation (6) (please also see nomenclature).

Figure 2 and Figure 3 show the plot of the entropy rate density as a function of the alloy parameters (diffuseness) and the processing and for the MEPR model. The first and second derivatives w.r.t. to V at constant ζ and G_SLI_ indicate that the entropy generation rate will increase linearly with velocity unless solute partitioning into the liquid is allowed. When solute partitioning is possible, the entropy rate generation term indicates a maximum when plotted as a function of velocity (Figure 2). If no other interface configuration is feasible (those that display a higher entropy rate generation, such as a seaweed, jagged or fine tip interface), the interface will remain planar during growth. Note that ${\dot{\varphi }}_{\max}$ cannot be less than zero (second law of thermodynamics). This implies that regardless of the sign of G_SLI_, the critical ${\dot{\varphi }}_{\max}$ can only have a lower value of zero for a planar interface. Thus, a non-planar shape can always overtake a plane front morphology for a negative temperature gradient, or in other words a negative temperature gradient will always imply a breakdown into cells or other patterns (unless a high-velocity-plane front transition occurs [2]). Additionally, because cellular shapes with a diffuse interface are seemingly restricted by the bounds of entropy from the diffuseness of alternate shapes, additional configurational entropy production rate increases for complex features (e.g., dendrites) are feasible and so will always emerge as an alternative structure unless a very wide diffuse interface topographies are possible with no partitioning. This is a possible explanation for why well-defined cellular features are not commonly noted in microstructures, such as atomized powders that solidify with a negative temperature gradient. Figure 2 and Figure 3 show the entropy generation rate density as a function of various solidification features and collapsed parameters that are known to influence instability of a particular topography or morphology. Note the definitions of **B**, **M**, and **N** from Equations (4)–(6) and the nomenclature. When **B** becomes greater than or equal to **M**, then **N** is either zero or negative; consequently, the interface diffuseness becomes undefined. The maximum entropy generation rate density increases with the corresponding increase in diffuse interface thickness and falls only when the parameter **B** approaches 0.5 **M**. The growth of the interface can be steady when **N** is greater than one. When the temperature gradient is zero, the diffuse interface thickness becomes undefined, thus allowing k_eff_ to take on a high value closer to one. When **B** is equal to **M**, then **N** is zero, and ζ and ${\dot{\varphi }}_{\max}$ are both undefined. From the transition instability criterion defined above, the peak for ${\dot{\varphi }}_{\max}$ against velocity occurs when **M/B** (dimensionless) is equal to 2 (i.e., **M**/**N^0.5^**) is equal to $\left(\frac{\;2\Delta h_{\mathrm{sl}}}{\;\Delta \rho_{k}{\;T}_{m}^{2}}\right)$. Figure 2 in [1] shows the plot of the entropy generation rate as a function of the diffuseness. When **M** > **B**, then the number of pseudo-atomic layers present within the diffuse interface region is easily related to the driving force diffuseness in an almost linear manner [1]. Note that the deviation from linearity sets in at a lower *V*/*G_SLI_* as the concentration increases. At the condition where **M** ≥ **N** > 1, noted in Figure 2, a steady slope is observed, where the V/G_SLI_ ratio shows a strong effect on the number of pseudo-atomic-spacings [1]. As the condition for 1 > **N** > 0 is encountered, even a small change in the V/G_SLI_ ratio can lead to a rapid change in the number of pseudo-atomic spacings at the interface. Figure 3 shows the parabolic-like profile of the entropy generation rate density as a function of V/G_sli_. Both Figure 2 and Figure 3 indicate that a peak is noted in the entropy generation rate density for a planar interface, essentially giving other entropy producing morphologies a possibility to dominate over the plane front structure (whether diffuse or not). An example is shown in Figure 3 of how the entropy rate for a cellular pattern or a dendritic morphology may indicate transitions to those shapes. Note that an implication of the results in Figure 1 is that ΔT_SLI_ approaches ΔT_O_, but a morphological transition prevents the full attainment for this separation for the plane front (i.e., if the high velocity plane front condition is not encountered) [2,13,38,39,40].

## 3. Traditional Instability Models

### 3.1. The CUT Model

The first interface breakdown model was proposed qualitatively by Rutter and Chalmers [22], and then quantitatively described by Tiller, Rutter, Jackson, and Chalmers [19]. This model describes the interface instability (from planar to non-planar) as being enabled by a region of constitutionally undercooled liquid that forms ahead of the solid–liquid interface during growth from solute partitioning. For a binary alloy, the CUT criterion for instability is written as:

(10) $$D_{L}={\left(\frac{V\;}{G_{L}\;}\right)}_{C}\Delta T_{O}$$

where G_L_ (Km^−1^) is the temperature gradient in the liquid, D_L_ (m^2^s^−1^) is the solute diffusion coefficient in the liquid, and ΔT_O_ (K) is the equilibrium solidification range (T_l_ − T_S_) for a liquid at composition C_O_ (molem^−3^). Also, T_l_ (K) and T_S_ (K) are the equilibrium liquidus and solidus temperatures captured in equilibrium phase diagrams.

### 3.2. The LST Model

In 1964, Mullins and Sekerka [31] proposed the linear stability model (LST) that considered the stability of a planar interface to a perturbation of an infinitesimal amplitude. In this stability model, the interface is unstable if any wavelength of a sinusoidal perturbation grows, and conversely the interface is stable if none of the perturbations can grow (regardless of their wavelength and surface energy) can grow. This LST criterion gives the instability criterion for a binary material as:

(11) $$D_{L}={\left(\frac{V\;}{G_{L}\;}\right)}_{C}\frac{\Delta T_{O}\left(K_{s}+K_{L}\right)\;S\;}{\;2{\;K}_{L}}$$

where S (no units) is the Mullins–Sekerka stability constant [31], which is equal to one for low velocities; K_L_ and K_S_ (J m^−1^K^−1^s^−1^) are the thermal conductivities for the fully solid and the fully liquid states, respectively. Note that the CUT model in Equation (10) and LST model in Equation (11) converge for the limit of $K_{s}\sim =K_{L}$.

A study by Burgeon et al. [38] with in situ interface imaging in microgravity conditions prevalent during the ordering of a cellular array structure concluded that the cause of interface dynamics and breakdown are more than just an account of the undercooled liquid ahead of the interface. An experimental study by Inatomi et al. [39] also cast doubt on whether an undercooled liquid or solute pile-up ahead of the interface is always present. They have argued persuasively that none of the theories for breakdown may be correct. For an interface’s topographical instability in the case of facet prone materials, a strain accumulation model [34] has also been considered as describing the interface breakdown. However, Inatomi et al. [39] argue against a general strain model as the cause for the instability. In reference 1, the variables Z_CUT_ and Z_LST_ were developed as parameters that describe the deviations from the experimental values. For the conditions where the interface instability occurs at high velocities, especially for very low alloy composition materials or with very low temperature gradients [1], both the CUT and LST models lose even more predictive capability [1,18]. Additionally, it should be noted the CUT and LST models do not address the facet/non-facet transitions or diffuseness at a molecular level, which is easily treated by the MEPR model [1,2].

## 4. Comparison with Experiments for the Diffusion Coefficient Prediction

The experimentally reported values of D_L_ from non-solidification experiments for Pb–Sn alloys at different concentrations are reported in [18] and are summarized in Table 1. The experimental D_L_ values shown in Table 1 directly measured from non-solidification experiments are corrected by an Arrhenius-type correction for the liquidus temperature if the report is at a higher temperature than the liquidus [30,31,32,33,34,35,36,37,38,39,40]. However, note that these corrected numbers only impact the results in a minor way for the dilute alloy compositions considered. The results shown in Table 1 for the D_L_ predictions for both CUT and LST show consistent and significant deviation from experimental measurements, as pointed out by De Cheveigne et al. [26] and Bensah et al. [18]. From Table 1, we note that the MEPR model shows stronger predictive capability of D_L_ compared to CUT and LST for Pb–Sn alloys when compared with experimental values. However, even with the MEPR model, large deviations are noted for experimental conditions with a small temperature gradient. Pb–Sn alloys are known to have very wide diffuse interfaces [1,21,35,36,37]. A lower G_SLI_ dramatically influences the diffuse interface as noted above, and consequently the partition coefficient. The expectation that k_eff_ approaches one with increased interface diffuseness is a reasonable assumption for dilute Pb–Sn and icosahedral alloys with a diffuse interface [32,33,40,41,42,43,44,45,46,47,48]. Should k_eff_, therefore, change from 0.636 to 0.95 because of the low temperature gradient and diffuse interface, the D_L_ value that is calculated is shown to become much lower to match the experimental data for even these low solidification temperature- gradient experiments. The corrected values are also shown in italics in Table 1 in the highlighted part of the table. Regardless, it should be noted that the assumed change in k_eff_ to 0.95 is arbitrary and is only set to this number to illustrate the influence of the partition function on the calculated number.

## 5. Summary Discussions

Topographical and diffuse interface reconfigurations occur with a change in the solidification rate. In this article, we pursue the hypothesis that the interface configuration during solidification is determined by the maximum rate of entropy production in the region between a rigorous solid and rigorous liquid phase. We posit that when an interface begins to migrate, there are several stable configurations that are possible. These include atomically planar, diffuse-planar, facet non-planar, and cellular nonplanar configurations. The configuration and topographical condition that affords the maximum entropy production rate (MEPR) yields the most stable interface configuration. The principle of MEPR is applied to (1) describe atomically smooth and diffuse interfaces, (2) provide quantitative results for the diffuse interface thickness and the number of pseudo-atomic layers in the interface region, and (3) predict the transition from planar to a non-planar facet or non-facet cellular morphology as a function of solidification velocity or temperature gradient. The MEPR model provides for an assessment of the interface diffuseness at the breakdown condition. It also allows for the break down condition to be expressed in terms of the cooling rate and the entropy generation rate.

Numerous experimental investigations spanning sixty years have failed to comprehensively validate any of the existing solid–liquid interface (SLI) growth instability models. With the MEPR model for the first time, breakdown conditions are predicted with a fair degree of accuracy for several binary alloys, where no previous theoretical model had predictability. The model considers steady-state solidification at close-to-equilibrium and far-from-equilibrium conditions. For dilute Pb–Sn alloys, the MEPR model gives closer D_L_ predictions compared to the predictions made by the more traditional CUT and LST models. Regardless of the success of the model to date, it should be noted that the model remains untested for alloys with a significant amount of solute content.

## Figures and Tables

**Table 1:** A summary of results for *D_L_* in instability conditions for experimental breakdown compared with the value obtained from three instability models. Note that for MEPR, G_SLI_ was assumed to be equal to G_L_ for the calculations_._ The diffusion data and physical constant from [18] for Pb–Sn alloys is *Ks* = 33.6 (J/mKs), *K_L_* = 15.4 (J/mKs); the equilibrium partition coefficient is k = k_eff_ = 0.636, Tm = 600.65 K, Δ*h_sl_ =* 2.48 × 10^8^ (J/m^3^). The shaded cells contain values of D_L_ (in italics) for both cases (i.e., the equilibrium partition ration as well as when k_eff_ increases to 0.95 for the low temperature gradient conditions). MEPR Model: Circa 2011; LST Model: Circa 1964; CUT Model: Circa 1953.

Composition	D_L_ (×10^−9^ m^2^/s) at T_l_ from Experiment *	*Imposed*	*Variable*	*Alloy*	*Variable*	k = 0.636 k = 0.95	D_L_ (×10^−9^ m^2^/s) at T_S_ from Solidification Theories		
		V_C_ (m/s) ×10^−6^	G_L_ (K/m)	Ts (K)	Tl (K)	MEPR Model Lower Bound	MEPR Model Upper Bound	CUT Model	LST Model
**Pb-0.01 *wt%* Sn**	1.656	167	540	600.621	600.604	4.619	2.3095	5.398	8.587
**Pb-0.03 *wt%* Sn**	1.655	73.5	820	600.560	600.508	4.016	2.008	4.692	7.465
**Pb-0.05 *wt%* Sn**	1.654	73.5	1380	600.499	600.412	3.976	1.988	4.646	7.392
**Pb-0.06 *wt%* Sn**	1.654	75.0	1220	600.469	600.364	5.507	2.7535	6.435	10.24
**Pb-0.1 *wt%* Sn**	1.653	56.7	1200	600.347	600.172	7.053	3.5265	8.241	13.11
**Pb-0.15 *wt%* Sn**	1.652	33.3	1300	600.194	599.933	5.733	2.8665	6.699	10.66
**Pb-0.15 *wt%* Sn**	1.652	108	415	600.194	599.933	58.24	29.12	68.06	108.3
						*2.97*	*1.485*		
**Pb-0.15 *wt%* Sn**	1.652	142	465	600.194	599.933	68.34	34.17	79.86	127.1
						*3.48*	*1.74*		
**Pb-0.15 *wt%* Sn**	1.652	167	485	600.194	599.933	77.06	38.53	90.05	143.3
						*3.93*	*1.96*		
**Pb-0.15 *wt%* Sn**	1.652	230	700	600.194	599.933	73.54	36.77	85.93	136.7
						*3.75*	*1.87*		

* Extrapolated to the liquidus temperature.

## Nomenclature and Abbreviations

**Letter symbols**	
Af	area of a solute flux in a liquid (m^2^)
A_SLI_	area of an interface in a solid–liquid region (m^2^)
B	Entropic contribution from the solute boundary layer
Cp	average heat capacity across a solid–liquid interface (Jm^−3^K^−1^)
d	interplanar lattice spacing (m)
$\Delta C_{O}$	change in concentration at a solute distance *z* (mole m^−3^) $\Delta C_{O}=\frac{\Delta T_{O}}{m_{L}}$
D	Diffusion Coefficient (m^2^s^−1^)
fs	fraction of liquid solidified at the solid–liquid interface (dimensionless)
G_S_	temperature gradient in a solid (Km^−1^)
G_L_	temperature gradient in a liquid (Km^−1^)
G_SLI_	linear temperature gradient across a diffuse interface (Km^−1^)
Δh_m_	heat of fusion of a solid with defects (Jm^−3^)
Δh_sl_	equilibrium heat of fusion (Jm^−3^) or (Jmol^−1^)
Js	solute flux in a liquid entering a solid–liquid interface (mole s^−1^)
k	equilibrium partition coefficient obtained from the phase diagram (dimensionless)
k_eff_	effective partition coefficient at a solid–liquid interface (dimensionless)
ΔKE	gain or loss in kinetic energy (J)
KL	thermal conductivity for a rigorous liquid (Jm^−1^K^−1^s^−1^)
KS	thermal conductivity for a rigorous solid (Jm^−1^K^−1^s^−1^)
m_L_	slope of the equilibrium liquidus line at the SLI for a binary material (Km^3^mole^−1^)
M	Entropic contribution from the thermal flow
N	Diffuseness contribution to the entropy generation
Q	lost work potential from the heat generation from a solid–liquid interface (J)
R_g_	molar gas constant (Jmol^−1^ K^−1^)
S	Mullins and Sekerka stability constant (dimensionless)
S_f_	flux entropy rate (JK^−1^s^−1^)
${\dot{s}}_{\mathrm{LG}}$	entropy generation density due to solute gradient in a liquid (Jm^−3^K^−1^)
${\dot{s}}_{\mathrm{SG}}$	entropy generation density due to solute gradient in a solid (Jm^−3^K^−1^)
${\dot{s}}_{E}$	change in entropy generation rate density due to exchange of matter and energy to and from a solid–liquid interface with its surrounding (Jm^−3^K^−1^s^−1^)
${\dot{s}}_{\mathrm{gen}}$	irreversible entropy generation rate in a diffuse region (JK^−1^s^−1^)
${\dot{s}}_{\mathrm{in}}$	rate of entropy entering a control volume (JK^−1^s^−1^)
${\dot{s}}_{\mathrm{out}}$	rate of entropy leaving a control volume (JK^−1^s^−1^)
${\dot{s}}_{\mathrm{gen}}$	total irreversible entropy generated rate density at an interface (Jm^−3^K^−1^)
${\dot{s}}_{\mathrm{LG}}$	entropy generation rate density by the solute gradient in a liquid (Jm^−3^K^−1^)
(𝑆𝑔𝑒𝑛)𝑚𝑎𝑥	maximum entropy generation due to lost work (JK^−1^)
𝑑𝑆_𝑐𝑣_⁄𝑑𝑡	total steady state entropy rate in a control volume (JK^−1^s^−1^)
𝑑𝑠_𝑐𝑣_⁄𝑑𝑡	total steady state entropy rate density in a control volume (Jm^−1^K^−1^s^−1^)
t	time (s)
Tli	liquidus temperature at a solid–liquid interface boundary (K)
Tsi	solidus temperature at a solid–liquid interface boundary (K)
ΔT_SLI_	temperature difference across a solid–liquid interface (K)
(𝑑𝐶𝐿𝐺⁄𝑑𝑧) or (𝛥𝐶𝑂⁄𝛿𝑐)	change in solute gradient in a liquid (mole m^−4^)
Tm	melting temperature (K)
Tav	average temperature between *Tli* and *Tsi* across a diffuse interface (K)
ΔT_O_	solidification temperature range (K)
V	solidification interface velocity (ms^−1^)
W_L_	lost work (J)
dz or δc	change in the position length of the solute (m)
Z_CUT_	deviation parameter of CUT from experiment at breakdown (dimensionless)
Z_LST_	deviation parameter of LST from experiment at breakdown (dimensionless)
**Greek symbols**	
Ωf	flux volume (m^3^)
ΔΩS	volume shrinkage (m^3^)
$\delta_{C}$	Solute boundary layer at the critical point $\delta_{C}=\frac{2\;D_{L}}{V}$ (m)
|Δρk|	density shrinkage (kgm^−3^) (*ρ*s- *ρl*)
*ρ*l	density of rigorous liquid (kgm^−3^)
*ρ*s	density of rigorous solid (kgm^−3^)
Δμ_c_	Chemical potential difference (Jmole^−1^)
ζ	solid–liquid interface thickness (m)
ω_D_	energy of defects (Jm^−3^)
Ω_SLI_	volume of a solid–liquid interface (m^3^)
$\dot{\varphi }$	maximum entropy generation rate density for a moving interface (Jm^−3^K^−1^s^−1^)
η_G_	driving force diffuseness (dimensionless)
η_T_	total diffuseness (dimensionless)
𝜂_𝛼_	thermal diffuseness (dimensionless)
**Subscripts and acronyms**	
CUT	constitutional undercooling theory circa 1953
LST	linear stability theory circa 1964
MEPR	maximum entropy production rate circa 2011
L	liquid
S	solid
LG	solute gradients in the liquid
SG	solute gradients in the solid
SLI	solid–liquid interface
HD	mean heat dissipation at the solid–liquid interface
f	facet
nf	non-facet
Expt	experiment
